# Bechterew’s Disease and the Risk of Spinal Fractures: Clinical Patterns, Imaging Correlation, and Outcomes

**DOI:** 10.7759/cureus.96260

**Published:** 2025-11-06

**Authors:** Kiranjot Kaur, Jideofor Okoye, William J Austin, Shenouda R Shehata Abdelmesih, Rana Ahmed, Shashwat Shetty, Shahmeen Rasul, Ayo-Oladapo Kolawole, Mariam Sabra, Kiran Ahmed

**Affiliations:** 1 US Navy, United States Military, North Chicago, USA; 2 Clinical Research, Arizona State University, Tempe, USA; 3 College of Medicine, Shri B. M. Patil Medical College, Vijayapura, IND; 4 Trauma and Orthopaedics, Mersey and West Lancashire Teaching Hospitals NHS Trust, England, GBR; 5 Orthopaedics, Queens Hospital Burton, Derby, GBR; 6 Orthopaedics and Traumatology, Royal Gwent Hospital, Newport, GBR; 7 Emergency Medicine, Hillingdon Hospitals NHS Foundation Trust, Uxbridge, GBR; 8 Orthopaedics, Hillingdon Hospitals NHS Foundation Trust, Uxbridge, GBR; 9 Trauma and Orthopaedics, University Hospitals of Derby and Burton (UHDB), Burton-on-Trent, GBR; 10 Orthopaedics, Surgical Interest Group, Africa, Lagos, NGA; 11 General Medicine, Alexandria University Faculty of Medicine, Alexandria, EGY; 12 Orhtopedics, Jinnah Postgraduate Medical Centre, Karachi, PAK

**Keywords:** bechterew’s sign, bechterew’s test, diagnostic accuracy, neurological assessment, spinal fractures

## Abstract

Bechterew’s disease, or ankylosing spondylitis (AS), is a chronic inflammatory spondyloarthropathy that causes spinal rigidity and increases the risk of unstable fractures, often after low-energy trauma. This systematic review included seven studies encompassing 672 patients with ankylosed spines who sustained spinal fractures. Clinical presentation commonly involves sudden back or neck pain, kyphotic deformity, limited spinal mobility, and neurological deficits, which may be subtle and easily overlooked. Fractures predominantly affect the cervical spine (C5-C7) and thoracolumbar junction (T11-L2), often extending through all three spinal columns, resulting in high instability and risk of spinal cord injury. Accurate imaging is critical; computed tomography delineates bony injuries, while magnetic resonance imaging identifies spinal cord damage, ligamentous disruption, and epidural hematomas. Nonoperative management carries a high risk of secondary displacement and neurological deterioration, whereas early surgical stabilization, typically via posterior or combined anterior-posterior fixation, improves outcomes. Multidisciplinary care involving orthopedic, neurosurgical, and critical care teams is essential for optimizing recovery. Limitations of the current literature include small sample sizes and heterogeneous study designs. Future research should focus on prospective multicenter studies, standardized imaging and management protocols, and long-term functional outcomes to reduce fracture risk and improve care in this high-risk population.

## Introduction and background

Bechterew’s disease, more widely recognized as ankylosing spondylitis (AS), is a chronic, progressive, and systemic inflammatory spondyloarthropathy that primarily affects the axial skeleton, leading to ossification of spinal ligaments, intervertebral joints, and entheses [1]. This pathological ossification produces the characteristic “bamboo spine” appearance on imaging and results in marked spinal rigidity and loss of flexibility. The disease predominantly affects young to middle-aged males, with a global prevalence of approximately 0.1-1.4%, varying by ethnicity and HLA-B27 gene prevalence [2]. The rigid and brittle nature of the ankylosed spine predisposes these patients to unstable vertebral fractures, even after minor or low-energy trauma such as falls from standing height. The incidence of spinal fractures in AS is estimated to be four to eight times higher than in the general population, with a reported lifetime fracture risk of up to 14% [3]. The majority of these injuries involve all three spinal columns, making them inherently unstable and prone to secondary displacement and neurological deterioration.

Clinically, patients may present with a sudden onset of neck or back pain following trivial trauma, often with a limited range of motion, kyphotic deformity, or neurological deficits such as weakness or sensory changes. However, the presentation may be subtle and easily overlooked, especially in the presence of preexisting spinal deformity or chronic pain typical of AS. Imaging plays a pivotal role in the diagnosis and evaluation of spinal fractures in Bechterew’s disease. Plain radiographs, while often the first-line investigation, are inadequate due to overlapping ossified structures and postural deformities. Computed tomography (CT) has emerged as the gold standard for detecting osseous injury and defining fracture morphology, while magnetic resonance imaging (MRI) is essential for identifying spinal cord injury, ligamentous disruption, epidural hematoma, and soft tissue involvement [4]. Given the tendency for multilevel and noncontiguous fractures, whole-spine CT or MRI is recommended in all suspected cases.

Fractures in Bechterew’s disease most frequently occur in the cervical spine, particularly at the C5-C7 levels, due to the combination of mobility and preexisting kyphosis in this region. The thoracolumbar junction (T11-L2) is the second most common site, accounting for a significant proportion of unstable three-column fractures [5]. Because the entire ankylosed spine behaves biomechanically as a long lever arm, forces from even minor impacts can lead to transverse fractures extending through all spinal elements. Neurological complications are common and serious. Studies have shown that up to 34-66% of AS-related spinal fractures are associated with spinal cord injury (SCI), and delayed or missed diagnosis significantly worsens outcomes. These fractures can lead to paralysis, respiratory compromise, and high mortality rates, particularly in elderly patients with comorbidities. Mortality following spinal fracture in AS has been reported to range from 15% to 32%, substantially higher than that of non-ankylosed trauma populations [6].

Clinical assessment should include neurological evaluation, spinal alignment examination, and a comprehensive trauma assessment, as secondary injuries are common. The use of Bechterew’s test, also known as the Sitting Straight Leg Raise (SLR) test, is a clinical examination maneuver used to assess radicular pain, particularly along the sciatic nerve distribution. The test is performed with the patient seated upright, during which one or both legs are actively extended at the knee, and may provide diagnostic clues, but is not specific for fractures [7]. Management depends on fracture stability, neurologic status, and patient comorbidity. However, conservative management carries a high risk of secondary displacement and delayed neurological deterioration. Therefore, early surgical stabilization is often via posterior or combined anterior-posterior fixation, which is generally recommended for most patients with unstable fractures. Non-operative management is reserved for medically unfit patients or stable injuries. Multidisciplinary care, involving orthopedic, neurosurgical, and critical care teams, is essential for optimizing outcomes and preventing complications.

The aim of this study is to evaluate the relationship between Bechterew’s disease (AS) and the increased risk of spinal fractures, with particular emphasis on identifying characteristic clinical patterns, imaging findings, and associated outcomes. This review seeks to highlight how the structural changes in the ankylosed spine predispose patients to fractures even with minimal trauma, assess the diagnostic value of imaging modalities such as MRI and CT in detecting these injuries, and analyze post-fracture complications and prognostic outcomes in this population.

## Review

Materials and methods

Search Strategy

A comprehensive literature search was systematically performed across four major electronic databases, namely, PubMed, Embase, Scopus, and the Cochrane Library, to identify relevant studies published between 2000 and 2024, following the Preferred Reporting Items for Systematic Reviews and Meta-Analyses (PRISMA) 2020 guidelines for systematic reviews and meta-analyses [8]. The search strategy incorporated the following key terms and their combinations: “Bechterew’s disease”, “ankylosing spondylitis”, “spinal fracture", “neurological injury”, and “imaging”. Boolean operators and MeSH terms were utilized to enhance search sensitivity. Only English-language studies were included, and reference lists of selected articles were manually screened to identify additional relevant publications.

Eligibility Criteria

Studies were included if they met the PICO framework criteria [9]: Population (P): patients with AS or related ankylosed spinal disorders; Intervention (I): clinical assessment, imaging evaluation, or diagnostic tests for spinal fractures; Comparator (C): standard trauma populations, non-AS patients, or alternative diagnostic approaches where applicable; Outcomes (O): incidence and pattern of spinal fractures, neurological deficits, imaging findings, management strategies, and clinical outcomes. Eligible studies encompassed original research, systematic or narrative reviews, and case series involving human subjects. Studies were excluded if they were animal-based, single case reports lacking imaging data, editorials, or conference abstracts without sufficient methodological detail or outcome reporting.

Study Selection and Data Extraction

Two independent reviewers conducted a structured screening of titles, abstracts, and full-text articles in accordance with the inclusion criteria. Discrepancies were resolved by consensus or consultation with a third reviewer. Data extraction focused on key variables including study design, population characteristics, diagnostic modalities (clinical and imaging-based), fracture site, neurological outcomes, and management approaches. Extracted data were synthesized in tabular form for clarity and comparative analysis.

Risk-of-Bias Assessment

The methodological quality of included studies was rigorously evaluated using appropriate standardized tools tailored to study design. Systematic and narrative reviews were assessed using the ROBIS (Risk of Bias in Systematic Reviews) tool, which evaluates bias across domains such as study identification, selection, and synthesis [10]. Cohort and observational studies were appraised with the Newcastle-Ottawa Scale (NOS), assessing participant selection, comparability, and outcome ascertainment [11]. Case reports and series were evaluated using the Joanna Briggs Institute (JBI) Critical Appraisal Checklist, ensuring transparency and validity in patient selection, diagnostic accuracy, and outcome reporting [12]. Each study was independently rated as having a low, moderate, or high risk of bias based on these criteria.

Data Synthesis

Extracted data were synthesized qualitatively and quantitatively where possible, following the PRISMA 2020 guidelines. Study characteristics, patient demographics, fracture patterns, imaging modalities, and clinical outcomes were tabulated to allow direct comparison across studies. Given the heterogeneity of study designs, populations, and outcome measures, a meta-analysis was not feasible; instead, a narrative synthesis was performed to summarize key findings. Trends in fracture location, associated neurological injury, diagnostic accuracy of imaging modalities, and management strategies were identified and critically analyzed. Emphasis was placed on highlighting patterns that could inform clinical decision-making and future research in patients with ankylosed spines.

Results

Study Selection Process

**Figure 1:**
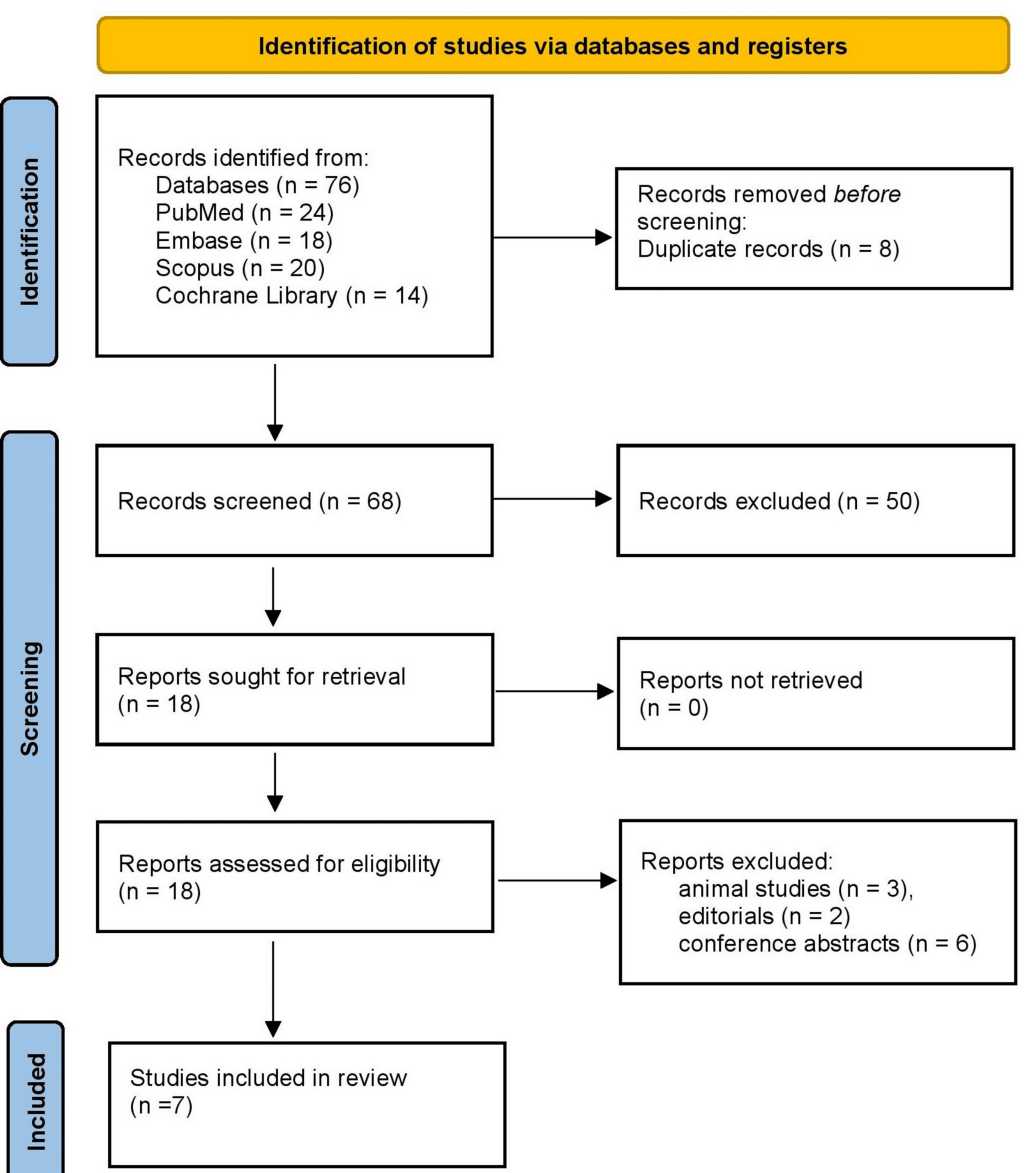
Preferred Reporting Items for Systematic Reviews and Meta-Analyses (PRISMA) 2020 flow diagram

Figure 1 shows that a total of 76 records were initially identified through database searching, including PubMed (n = 24), Embase (n = 18), Scopus (n = 20), and the Cochrane Library (n = 14). After removing eight duplicate records, 68 records were screened based on titles and abstracts, and 50 records were excluded for not meeting the inclusion criteria. Full-text articles were retrieved for 18 records, of which 11 studies were further excluded due to reasons such as animal studies (n = 3), editorials (n = 2), and conference abstracts (n = 6). Finally, seven studies met all eligibility criteria and were included in the systematic review. This stepwise selection process, in accordance with PRISMA 2020 guidelines, ensured that only relevant studies focusing on spinal fractures in patients with AS or related ankylosed spinal disorders were included for qualitative synthesis.

Characteristics of the Selected Studies

Table 1 summarizes seven studies on spinal fractures in ankylosed spines. Fractures were often missed on plain radiographs, with CT and MRI recommended, mainly affecting cervical and thoracolumbar regions [13]. AS patients had higher fracture incidence, complications, and mortality [14], with ~34% experiencing spinal cord injury, especially in cervical fractures [15]. Nonoperative management carries a high risk, emphasizing diagnostic and therapeutic algorithms [16]. Full-spine CT and MRI were recommended due to high displacement risk [17]. AS fractures had higher morbidity than diffuse idiopathic skeletal hyperostosis (DISH), mostly cervical [18]. Low-energy trauma could cause multiple thoracolumbar fractures (T11-T12) [19].

**Table 1:** Characteristics of the selected studies AS: ankylosing spondylitis, DISH: diffuse idiopathic skeletal hyperostosis, SCI: spinal cord injury, CT: computed tomography, MRI: magnetic resonance imaging

Author (Year)	Population	Intervention / clinical test used	Comparator	Outcome	Imaging used	Site of fracture
Westerveld et al. (2008) [13]	Literature review / pooled case series of patients with ankylosed spines	Clinical evaluation; emphasis on trauma evaluation and whole-spine assessment	General trauma population	Fractures often missed on plain films; worse outcomes vs non-ankylosed spines	Plain radiographs often insufficient → CT ± MRI recommended	Cervical and thoracolumbar
Lukasiewicz et al. (2016) [14]	National cohort of AS patients with spine fractures	Standard trauma exam; neurologic assessment	Non-AS trauma	Increased fracture incidence, complications, and mortality	CT and MRI used for diagnosis and cord injury assessment	Cervical
Teunissen et al. (2017) [15]	172 patients with ankylosed spine fractures (AS + DISH)	Neurologic exam; trauma assessment	Patients with vs without SCI	SCI in ~34%; cervical fractures had higher risk	CT for bony injury; MRI for cord and ligaments	Cervical
Reinhold et al. (2018) [16]	Review + case examples of ankylosed spine fractures	Diagnostic and therapeutic algorithm	Operative vs conservative strategies	Nonoperative treatment risky due to secondary deterioration	CT for bone detail; MRI for soft tissue/cord	Cervical, thoracolumbar
Werner et al. (2016) [17]	Review of spinal fractures in AS	Imaging-first trauma evaluation	General trauma	High fracture and displacement risk	CT and MRI; full-spine scans recommended	Cervical > thoracic/lumbar
Chen et al. (2023) [18]	Retrospective cohort: AS vs DISH fractures	Neurologic and radiologic assessment	AS vs. DISH comparison	Higher morbidity and mortality; differences in management	CT ± MRI for diagnosis and planning	Cervical
Samartzis et al. (2005) [19]	Case report: elderly AS patient with multiple fractures	Clinical exam, imaging after low-energy falls	Case report (no control)	Multiple fractures after minor trauma	X-ray → CT for characterization	Thoracolumbar (T11-T12)

Risk-of-Bias Assessment

Table 2 shows that the risk of bias across the included studies varied based on the study design. Westerveld et al. (2008), a systematic review and pooled case series, had a moderate risk due to heterogeneous studies and variable reporting [13]. Lukasiewicz et al. (2016), a retrospective national cohort, had a low risk with standardized coding and minimal selection bias [14]. Teunissen et al. (2017), a multicenter cohort, was a moderate risk due to retrospective design despite robust imaging confirmation [15]. Narrative reviews by Reinhold et al. (2018) and Werner et al. (2016) were high risk owing to non-systematic methodology and lack of predefined criteria [16,17]. Chen et al. (2023), a retrospective cohort, had low-moderate risk, limited by a single-center design [18]. The case report by Samartzis et al. (2005) was high risk, reflecting limited generalizability [19].

**Table 2:** Risk-of-bias assessment AS: ankylosing spondylitis, DISH: diffuse idiopathic skeletal hyperostosis, ROBIS: risk of bias in systematic reviews, NOS: Newcastle-Ottawa Scale, JBI: Joanna Briggs Institute, SCI: spinal cord injury

Study	Study Design	Risk of Bias Tool	Risk of Bias Rating	Justification
Westerveld et al. (2008) [13]	Systematic review / pooled case series	ROBIS (Risk of Bias in Systematic Reviews)	Moderate	Heterogeneous included studies with variable reporting; lacks uniform methodology though comprehensive in scope.
Lukasiewicz et al. (2016) [14]	Retrospective national database cohort	Newcastle–Ottawa Scale (NOS)	Low	Large national dataset with adjusted analyses and standardized coding; minimal selection bias.
Teunissen et al. (2017) [15]	Retrospective multicenter cohort	Newcastle–Ottawa Scale (NOS)	Moderate	Robust imaging confirmation, but retrospective design limits control over confounders.
Reinhold et al. (2018) [16]	Narrative review with case examples	ROBIS	High	Non-systematic methodology, expert opinion elements, potential publication bias.
Werner et al. (2016) [17]	Narrative review	ROBIS	High	Descriptive review without systematic search; absence of predefined inclusion/exclusion criteria.
Chen et al. (2023) [18]	Retrospective cohort (AS vs DISH)	Newcastle-Ottawa Scale (NOS)	Low-Moderate	Consistent imaging and outcome measures; single-center design introduces mild selection bias.
Samartzis et al. (2005) [19]	Case report	JBI Critical Appraisal Checklist for Case Reports	High	Single patient observation; lacks generalizability and external validity.

Discussion

Patients with Bechterew’s disease, also known as AS, are at a markedly increased risk of spinal fractures due to chronic inflammation, ligamentous ossification, and vertebral ankylosis, which render the spine rigid and brittle, predisposing it to injury even after low-energy trauma [13]. Clinically, patients may present with sudden onset of neck or back pain, reduced spinal mobility, kyphotic deformity, or neurological deficits such as weakness or sensory changes, which may vary depending on the level and severity of the fracture [14,15]. Cervical fractures, particularly at the C5 to C7 levels, are most common due to residual mobility and mechanical stress, while thoracolumbar fractures involving T11 to L2 occur frequently because of transition zones susceptible to three-column instability [13,16]. Low-energy trauma may result in multilevel or noncontiguous fractures, highlighting the distinctive clinical pattern of spinal injuries in Bechterew’s disease compared to non-ankylosed populations [14,19].

Accurate imaging correlation is essential for diagnosis and management. Plain radiographs are often insufficient due to overlapping ossified structures and postural deformities, whereas CT provides a detailed assessment of bony anatomy and fracture morphology, and MRI is critical for evaluating ligamentous injury, spinal cord damage, epidural hematoma, and soft tissue involvement [13,17]. Whole-spine imaging is recommended to detect multilevel or noncontiguous fractures, and comparative analyses between AS and diffuse idiopathic skeletal hyperostosis indicate that AS patients have higher fracture severity, greater neurological risk, and increased mortality, necessitating disease-specific imaging and management protocols [18].

Spinal fractures in Bechterew’s disease are associated with high rates of spinal cord injury, reported in approximately 34-66% of cases, and delayed or missed diagnosis substantially worsens prognosis [15,18]. Mortality after spinal fracture ranges from 15 to 32 percent, which is significantly higher than in non-ankylosed trauma populations [6]. Nonoperative management carries a high risk of secondary displacement and neurological deterioration, emphasizing the need for early surgical stabilization, typically via posterior or combined anterior-posterior fixation, to restore spinal stability and protect neurological function [20]. Multidisciplinary care involving orthopedic surgeons, neurosurgeons, anesthesiologists, and critical care teams improves outcomes, reduces complications, and facilitates early rehabilitation [13,16].

The main limitations of the current literature include small sample sizes and retrospective study designs with heterogeneous fracture classification and imaging protocols, which limit generalizability and standardization. Future research should focus on prospective multicenter studies with standardized imaging and management protocols, development of predictive tools for fracture risk stratification, long-term evaluation of functional outcomes and quality of life, and assessment of minimally invasive stabilization techniques and pharmacologic interventions to reduce fracture risk.

## Conclusions

Bechterew’s disease, or AS, significantly increases the risk of spinal fractures due to chronic spinal rigidity, ligamentous ossification, and altered biomechanics. These fractures most commonly occur in the cervical and thoracolumbar regions and often result from low-energy trauma. Clinical patterns frequently include sudden onset of back or neck pain, spinal deformity, and neurological deficits, which can be subtle and easily overlooked. Imaging correlation using computed tomography and magnetic resonance imaging is essential for accurate diagnosis, detection of multilevel injuries, and assessment of spinal cord involvement. Fractures in this population are associated with high rates of spinal cord injury, secondary displacement, and mortality, emphasizing the need for early recognition and timely surgical stabilization. Multidisciplinary management remains crucial to optimize outcomes, prevent complications, and facilitate rehabilitation. Future research should focus on standardized diagnostic and treatment protocols, risk stratification models, and long-term functional outcomes to improve care for patients with ankylosed spines.
